# Preparing for the “black swan”: Reducing employee burnout in the hospitality sector through ethical leadership

**DOI:** 10.3389/fpsyg.2022.1009785

**Published:** 2022-10-12

**Authors:** Anis Ali, Tasawar Abdul Hamid, Rana Tahir Naveed, Irfan Siddique, Hyungseo Bobby Ryu, Heesup Han

**Affiliations:** ^1^Department of Management, College of Business Administration, Prince Sattam Bin Abdulaziz University, Al-Kharj, Saudi Arabia; ^2^OUS Royal Academy of Economics and Technology in Switzerland, Zurich, Switzerland; ^3^Division of Management and Administrative Sciences, University of Education (UE) Business School, University of Education, Lahore, Pakistan; ^4^Faculty of Management Studies, University of Central Punjab, Lahore, Pakistan; ^5^Food Franchise Department, College of Health Sciences, Kyungnam University, Changwon, South Korea; ^6^College of Hospitality and Tourism Management, Sejong University, Seoul, South Korea

**Keywords:** ethical leadership, burnout, resilience, hospitality, subjective wellbeing

## Abstract

Hospitality is at a crossroads. While the growth and developmental indicators in this sector show economic potential, the rising employee burnout rate is a serious challenge to hospitality management. Literature suggests that an ethical leader can reduce employee burnout significantly. Although hospitality employees face a higher risk of burnout than other service segments, shockingly, past leadership studies did not focus on how ethical leaders in a hospitality organization may reduce the risk of burnout. Therefore, we conducted this research to explore ethical leadership-burnout relationships in the hospitality sector with the mediating effects of subjective wellbeing and employee resilience. A questionnaire was provided to employees in different hotel organizations (*n* = 346). Structural equation modeling was employed for hypothesis testing. The statistical evidence supported the theoretical assumptions that ethical leadership negatively predicts employee burnout, and subjective wellbeing and resilience mediate this relationship. The outcomes of this study suggest different theoretical and social implications. For example, the findings indicate the effectiveness of ethical leadership in reducing employee burnout in the hospitality sector. Several other implications have been discussed in detail.

## Introduction

Employee burnout has emerged as one of the pressing issues for many contemporary organizations worldwide, with many negative outcomes (Gharakhani and Zaferanchi, 2019). A recent estimate by World Economic Forum suggests that the global economic cost of employee burnout is more than 300 billion USD which is almost greater than the combined financial gains of the top 10 multinationals, including Apple and Alphabet (Bruce, 2019). World Health Organization (WHO) specifies burnout as a syndrome that is related to unmanaged workplace stress (World Health Organization., 2019). Current evidence by GALLUP on employee burnout indicates that almost 76% feel that they are burnout sometimes, and around 30% feel that they are burned out almost all the time (Ben, 2020). The same GALLUP document shows that more than 60% of employees, who feel that they are burned out, are more likely to take sick leaves and even think of quitting their jobs. This number is alarmingly high, indicating that employee burnout is a critical challenge for organizational management.

Excessive workload, blurred job nature, and stressful working conditions have been identified as risers of employee burnout. However, regrettably, due to a dynamic business environment, stiff competitiveness, and several other factors (Deng et al., 2022), employees face pressure in working situations more often than ever. Contemporary employers expect employees to assume more diverse work responsibilities, negatively affecting their mental health. Given that most organizations expect their employees to be more and more productive, burnout may exist in any segment of an economy (Naz et al., 2016; Asghar et al., 2019). Nonetheless, the hospitality sector faces more employee burnout risk than other service segments (Lederer et al., 2017; Nawaz and Sandhu, 2018). Indeed, hospitality employees are expected to be exposed to demanding consumers and difficult situations. Further, increasing workload, irregular working hours, chronic stress and the 24/7 working nature of this industry may lead employees to face the risk of burnout (Andrew et al., 2022).

Although the issue of employee burnout is a common workplace phenomenon, most companies tend to relate it as a personal issue that is misleading. Buttressing this, Rothstein (2021) indicated that burnout is a challenge that relates to the organization, not the individuals, implying that leadership in an organization has a clear role in reducing employee burnout (Dyrbye et al., 2020; Kelly and Hearld, 2020). The top five factors that correlate with employee burnout, as specified in a recent GALLUP survey (unfair treatment, workload, unclear communication, managerial support, and time pressure), all relate to leadership (Kelly and Hearld, 2020). Corporate leaders should be responsible for protecting their employees against unfair treatment and workload. In addition, they should provide the necessary support to the employees to reduce burnout risk. Past literature specified different leadership approaches for effective organizational management, including transformational leadership (Eliyana and Ma'arif, 2019; Deng et al., 2022), servant leadership (Ahmad et al., 2021a; Peng et al., 2022), ethical leadership (Ahmad et al., 2018; Murtaza et al., 2021), and others. Although different leadership styles have been discussed previously, most cases examined the positive effect of a certain leadership style. For instance, the role of ethical leadership has been mentioned to influence different employee outcomes, including creativity (Kalyar et al., 2020) and organizational innovation (Shafique et al., 2020), organizational learning (Usman and Hameed, 2017) and exploitative and explorative learning (Ali et al., 2022). Recently, some behavioral management scientists have proposed the role of leadership, especially ethical leadership, to deal with negative work-related outcomes, for example, how the manifestation of an ethical leader could reduce the knowledge hiding in an organization (Abdullah et al., 2019; Anser et al., 2021), and burnout (Franczukowska et al., 2021; McKenna and Jeske, 2021). Shockingly, literature on ethical leadership and its association with burnout is sparse in hospitality management. Therefore, this research examines the effect of an ethical leadership style on employee burnout in hospitality management.

Different psychological factors predict employee burnout in an organizational setting. For example, stress and work alienation as mediators to predict employee burnout has been discussed in prior literature (Parveen and Adeinat, 2019; Usman et al., 2020). Similarly, psychological contract violation (Ali et al., 2019), emotional exhaustion (Arshadi and Shahbazi, 2013), role conflict (Schaufeli et al., 2009) and role clarity (Chen et al., 2022) were identified as significant mediators to explain employee burnout. From a positive psychology perspective, the literature suggests that subjective wellbeing perceptions of employees with respect to their employer and resilience could mediate employee burnout (Yu and Chae, 2020; Lan et al., 2021). As per the definition of Yu and Chae (2020), subjective wellbeing is the rational and affective evaluation of an employee for his/her employer. Denovan and Macaskill (2017) indicated that subjective wellbeing is equally important to improve employees' mental health and reduce burnout risk. According to Laura (2022), burnout exists in an organization when employees experience a low wellbeing situation because of unmanaged workplace stressors. According to a recent study most of the employees think that their managers matters most to improve their wellbeing in a workplace (Limeade., 2016). Undoubtedly, the role of corporate leaders, especially ethical leaders, in ensuring employee wellbeing is of utmost importance to reduce burnout risk (Vullinghs et al., 2020). This clearly explains how wellbeing, as an outcome of ethical leadership, can explain burnout as a mediator. Resilience, on the other hand, is a psychological element of an employee to face and get rid of extreme workplace situations (Zautra et al., 2010). The scholars like Shin et al. (2012) have argued that resilience explains a mechanism to adjust to uncertain working conditions in an organization. Resilience helps employees to fight against burnout in the workplace (Kutluturkan et al., 2016). Leadership scholars believe that corporate leaders significantly influence employee resilience (Nguyen et al., 2016). Specifically, it has been mentioned in the prior literature that an ethical leader manifests different personnel management strategies to improve the resilience level of employees (MacIntyre et al., 2013a). An ethical leader develops interpersonal linkages with employees which creates a positive working environment, ultimately improving the resilience level of employees. Indeed, Uppathampracha (2022) indicated that ethical leadership influences employee resilience positively which then explains employee creativity. Because, ethical leaders can influence employee resilience, and because resilience may explain burnout, we believe there is a mediating role of employee resilience between ethical leadership and burnout.

Although the mediating effects of subjective wellbeing and resilience to reduce employee burnout were mentioned in the literature, in a leadership framework (Hayat Bhatti et al., 2020; Chen et al., 2022), such studies are underexplored. We feel it worthwhile to investigate such mediating effect, as an outcome of ethical leadership, due to two important reasons. First, recent evidence suggests that corporate leaders play a pivotal role in improving employees' wellbeing by providing them with the necessary resources (Gregersen et al., 2016). This indicates that the leadership in an organization has a definite role in influencing employee resilience, which can then reduce their burnout. Therefore, advancing this debate in an ethical leadership framework is important. Second, although there have been studies mentioning that resilience is influenced by genetic factors (Leys et al., 2020). However, some recent researchers mentioned that resilience is a dynamic personal capability that can be learned, developed and influenced by different social and contextual factors (leadership, for example) (Cooper et al., 2019). Same kind of observation was shared by Riopel (2019). Specifically, leaders are in great positions to influence employee resilience. Therefore, it is worthwhile to investigate the mediating effect of resilience in reducing employee burnout in an ethical leadership framework.

All in all, this research intends to bridge the following knowledge gaps. First, this is the first study that considers the leadership aspect, especially ethical leadership style, from a burnout standpoint. To this end, most of the leadership literature emphasizes the positive aspect of a leadership style (Fang et al., 2019; Islam et al., 2021), whereas how a certain leadership style relates to burnout was less investigated earlier. Second, this study advances the debate on mitigating employee burnout by proposing a robust model because it considers both the organizational factors (leadership) and psychological factors to explain employee burnout. Third, this study attempts to highlight the critical challenge of employee burnout in the hospitality sector from a developing country's standpoint, which was not focused on earlier because most investigations were conducted in developed or high-income nations (Cocchiara et al., 2019; Montgomery et al., 2019). Employees in developing countries face more difficult working and social conditions due to resource scarcity (Ashkar et al., 2010). Though some recent researchers investigated burnout in a developing country like Pakistan (Salama et al., 2022), however this sparse explanation is insufficient. Therefore, investigating employee burnout, as an antecedent of ethical leadership, from the standpoint of a developing country is important.

The remainder of this study has been arranged in four major parts including theory and hypotheses, methodology, results and discussion. In theory and hypotheses part, we have discussed the related literature and the underpinning theory of this research. Similarly, the methodology part relates to the process of data collection, sampling and instrument, etc. The results section describes how the proposed relationship were validated by opting different statistical tools. Lastly, the discussion section highlights the major finding with respect to previous studies. We have also discussed different implications as well in the same section followed by conclusion.

## Theory and hypotheses

To justify the theoretical reasoning, we used the conservation of resources (COR) as the underpinning theory which was introduced in 1989 by Hoboll. He argued that people are expected to attain, build, and protect different valuable resources which can shelter them in uncertain situations (Hobfoll, 1989). Halbesleben et al. (2014) provided an academic definition of resources by stating that resources may be anything (personal or contextual) that individuals feel is valuable for completing a certain task. In general, literature discusses two major aspects of resources under this theory. One aspect focuses on resource sufficiency (value addition) the other aspect views resources from a depletion or loss perspective (insufficiency). The prior viewpoint of resources indicates that people in an organization have sufficient resources. Such people are less likely to face a resource bleeding situation to shelter them against extreme situations. Hence, people with better resource availability are expected to show better energy, commitment and motivation level in completing a task (Ng and Feldman, 2012). The latter viewpoint of resources under COR indicates that people face a resource scarcity or loss situation in an organization, creating a stressful situation for them that ultimately determines burnout. Drawing upon COR, we suggest that hospitality employees often face stressful situations and if the employers do not help employees, they feel a resource loss situation, which gives rise to burnout. Reflecting this, Hobfoll (2001) believed that in stressful working conditions, when employees feel they have insufficient resources (especially contextual resources) or they feel their resources are lost while fixing a certain situation, this feeling of resource loss or insufficiency leads them to face the risk of burnout. To recover from different negativities in a workplace, employees need contextual resources. This clearly specifies the role of leadership. Piccolo and Colquitt (2006) were of the view that corporate leaders play an important role for employees to recover from a resource loss or insufficiency situation as an influential leader not only facilitates employees with needed resources, but he or she also supports/facilitates employees by guiding, encouraging and praising them in different situations. Past researchers have also employed COR theory from an employee burnout perspective (Lu and Guy, 2019; Akirmak and Ayla, 2021). Even leadership scholars have found this theory helps to explain employee burnout in a leadership framework (Afshan et al., 2021). This is why we believe this theory has the potential to explain our theoretical arguments with respect to the current context.

In general, literature mentions employee burnout on dimensions like de-personalization, emotional exhaustion, and low personal accomplishment (Maslach, 2003; Halbesleben, 2006). To this end, corporate leaders can influence different employee outcomes by providing different resources to the employees, which limits the burnout risk of employees (Kanste et al., 2007; Arnold et al., 2015). Especially ethical leadership style to predict employee burnout was recently discussed (Mo and Shi, 2017; Ali et al., 2021).

Brown et al. defined ethical leadership as a style of organizational management in which a corporate leader exhibits normative ethical behavior with the employees by developing interpersonal associations and considering their input in organizational decision-making (Brown et al., 2005). Specifically, an ethical leader tends to enhance employees' wellbeing by solving their problems and providing them with a flexible and positive work environment (Franczukowska et al., 2021). A positive, supportive and flexible working environment developed by an ethical leader is important to employees' mental health, which ultimately serves as a contextual resource reducing the likelihood of employee burnout. On a further note, an ethical leader is expected to affect different employee outcomes through his or her ethical conduct, which improves the mental health of employees on one end, and prevents employees from experiencing the risk of burnout (Eslamieh and Mohammad Davoudi, 2016). Similarly, the central focus of an ethical leader is on altruism and ethical support and guidance to the employees without prejudice (Brown and Treviño, 2006). Therefore:

H1: An ethical style of leadership negatively relates to employee burnout.

The concept of ethical leadership relates well to positive employee psychology literature. A positive link between ethical leadership and employees' subjective wellbeing has been repeatedly discussed in prior literature (Yang, 2014; Kaffashpoor and Sadeghian, 2020). Specifically, the literature argues that an ethical leader enhances employees' mental health by focusing on their issues and facilitating them in achieving different organizational tasks (Li et al., 2014). Moreover, an ethical leader is one who treats employees fairly. When employees receive fair treatment, it boosts their morale and mental health, which then fosters their subjective wellbeing (Tu et al., 2017). Such leaders intend to provide the employees with optimal workplace settings by inspiring, building and maintaining an environment of trust and respect. Employees positively evaluate these steps of their ethical leaders, which then improve their subjective wellbeing (Sarwar et al., 2020). In addition, an ethical leader strongly focuses on ethics and morality, which fosters the overall better moods of employees and converts them into happy workers (Yang, 2014). Precisely, the role of an ethical leader for an organization is like roots, and in line with the findings by Giulia (2021), when roots are solid, the stress and tension on the part of employees will be short-lived.

Research on employee psychology indicates that subjective wellbeing improves employees' mental health (Singhal and Rastogi, 2018; Salgado et al., 2019). Organizational scientists have provided empirical and theoretical evidence that employees' empirical and theoretical evidence that employees' subjective wellbeing can reduce workplace negativities (Katana et al., 2019; Darvishmotevali and Ali, 2020). Further, past literature acknowledges the mediating effect of subjective wellbeing. For example, from the standpoint of different workplace stressors, the mediating role of subjective wellbeing was discussed in prior literature (Gordon et al., 2018; Yildirim and Arslan, 2020). Even in an ethical leadership framework, the mediating role of subjective wellbeing exists (Hayat Bhatti et al., 2020). All in all, as ethical leadership positively predicts subjective wellbeing, and because subjective wellbeing could be a significant mediator in predicting different employee outcomes, we expect:

H2: An ethical leadership style positively relates to the subjective wellbeing of employees.

H3: Subjective wellbeing mediates between ethical leadership and employee burnout.

Research suggests that employee resilience is critical in predicting different employee outcomes in an organizational setting, including emotional wellbeing and professional success (Brennan, 2017). Richardson (2002) and Thies and Travers (2006) were pioneers who extended the debate on resilience from an organizational perspective by stating that it is a dynamic capability of a person to adapt to uncertain workplace situations. Indeed, King et al. (2016) have argued that a person's resilience enables him or her to find and arrange resources (personal and contextual) to eliminate the negativities pertinent to a workplace. They further stressed that resilience also helps individuals to regain equilibrium. Research on employee resilience has been flourishing recently. Stiff rivalry, pressure situations, irregular working hours, and blurred lines between employees' personal and professional lives urge contemporary scholars to study employee resilience more and more (Sandifer and Walker, 2018; Lupe et al., 2020). The early researchers emphasized that resilience relates to the genetics of an individual. However, some recent behavioral scientists mentioned that resilience could be learned and developed (Riopel, 2019). From this perspective, corporate leaders have a definite role in influencing employee resilience (Harland et al., 2005; Nguyen et al., 2016). Specifically, the positive steps taken by an ethical leader for employees' wellbeing are expected to influence employees' cognition and satisfaction. Moon and Jung (2018) indicated that the moral character of a corporate leader focuses on fostering employees' mental health through a positive and healthy work environment. MacIntyre et al. (2013b) believed that ethical leaders can influence the resilience of their followers by building respect, consistency and trust, which then influences their resilience.

Literature suggests that employees' resilience negatively predicts burnout in an organizational setting (Kutluturkan et al., 2016; West et al., 2020). Resilience helps an employee to bounce back against the negative effect of workplace stress, and thereby, it serves as a protecting factor again employee burnout (Taku, 2014). From a leadership perspective, a leader helps employees to build a higher level of resilience by promoting employees' mindfulness and personal resilience plans (Sommer et al., 2016). Moreover, the ethical focus of a leader on the employees enriches their trust and satisfaction, which leads employees to a greater resilience level. Ultimately this process explains how ethical leadership predicts employee burnout. Therefore:

H4: It is expected that ethical leader positively influences employee resilience.

H5: Resilience mediates between ethical leadership and burnout.

## Methods

### Data collection

This study chooses the hospitality sector of Pakistan. This South Asian country has grown significantly in tourism and hospitality. Known for different tourists locations, this country has been recognized as “the best holiday destination” in the recent past (DAWN., 2019). Tourist numbers massively increased in the recent past (more than 300%) (Ikram, 2019). Current data suggest that there will be a 30% expected increase in international tourists by 2030 (Khurshid, 2019). Although the growth and developmental evidence in this sector are strong, unfortunately, employees in this sector are more prone to the risk of burnout compared to other service segments. Compared to the developed or high-income countries, most developing countries' economic segments are notresource-abundant, due to which employees in such countries face more difficult situations, thereby increasing the risk of burnout. Therefore, reducing this risk in an ethical leadership framework is worthwhile.

For data collection, we selected different hotels (large hotel enterprises) in Lahore and Islamabad. The former is the capital of Punjab province, while the latter is the country's capital. Both cities are famous from the perspective of tourism and hospitality. This is why both national and international hotel chains exist in these two cities. We contacted different hotels to allow us to maintain direct interaction with their employees. Some hotels responded positively (four from Lahore and three from Islamabad) to our request. Such hotels were then approached (a schedule was pre-decided with the management). The data were gathered in two waves separated by a two weeks interval. In the first wave, we collected data on ethical leadership, subjective wellbeing and resilience (402 responses). In the second wave, the data on burnout was collected (374 responses). Specifically, it took two months to collect the data (February and March 2022).

### Instrument

We used a questionnaire for data collection in this survey. The responses were gathered from the employees on a 5-point Likert scale. In precise, the variable's items (ethical leadership- ETL, employee burnout-EBO, subjective wellbeing-SWB, and employee resilience-ERS) were taken from published material (for more detail, please see the coming section). These items were presented to the experts for evaluation of either these items were appropriate with respect to the theme of this study (Adnan et al., 2021; Awan et al., 2021; Guan et al., 2022). After the confirmation from the experts, the final instrument was given to the employees to receive their responses. The questionnaire consisted of different parts, for example, the first page included the informed consent information. Similarly, the other pages were related to socio-demographic (e.g., age, income, experience) and variable-related information. In addition, the guidelines of the Helsinki Declaration were followed (Alam et al., 2021; Ullah et al., 2021; Ahmad et al., 2022a).

### Sample size and data cleaning

The sample size was estimated through A-priori sample size calculator (Dniel, 2010), which shows that a sample size of 341 should be sufficient for this research. Previous researchers have also used this tool to determine the sample size (Valaei and Jiroudi, 2016; Yadav et al., 2018). We distributed 500 questionnaires among the respondents, and we received 374 filled responses. Among 374 responses, 28 were discarded because either these responses did not comprise the full information or were outliers. Finally, we included 346 valid responses.

Employees from different departments and ranks participated in this survey. Both male of female employees were included. The percentage of male respondents was higher (73%) than the female. This is justifiable as Pakistan is still considered a dominant male society (Ahmad et al., 2021b). The ages of most employees were between 18 to 40 years (79%), and the experience ranged between (most of the employees) 3–10 years.

### Measures

The items to measure ETL were taken from Brown et al. (2005), which included ten-items (“My manager/leader discusses business ethics or values with employees” and “My manager/leader sets an example of how to do things the right way in terms of ethics”). EBO was quantified through a seven-items developed by Kristensen et al. (2005). Some example items from this scale were “I feel worn out at the end of the working day” and “I am exhausted in the morning at the thought of another day at work” The mediating variables in this survey (SWB and ERS) were measured by using the scales of Lyubomirsky and Lepper (1999) and Smith et al. (2008), respectively. In precise, the scale of SWB consisted of four-items (“In general, I consider myself a very happy person”), and the scale of ERS consisted six-items (“I tend to bounce back quickly after hard times”). All adapted scales showed a significant reliability value (α = 0.87, 0.85, 0.80, and 0.88 for ETL, EBO, SWB, and ERS, respectively).

### Common method bias

Although we employed a two-wave data collection strategy to limit the issue of CMB, we still executed the famous technique of the common latent factor (CLF) test to detect CMB. To do this, two measured models were constructed in AMOS software. The first model was the original four-factor hypothesized with no inclusion of any CLF. The second model was a CLF-based model, which was allowed to influence all observed variables (a total of 27) in this study. The outputs of both models (the standardized regression weights) were then compared to detect any significant variance (>0.2). The results showed that, though both models differed, however, such differences were minor ones (<0.2), showing that a CLF did not produce any significant effect in the model. This was a statistical confirmation for the non-existence of CMB.

## Results

### Reliability and validity

After data collection, adjusting data cleaning issues and confirming the non-existence of CMB, we analyzed the data. To start with, we established the convergent validity and composite reliability (CR) for ETL, EBO, SWB, and ERS. The below formula given in Equation (1) was used to estimate average variance extracted (AVE) values. This formula calculates AVEs based on the standardized factor loadings of a specific variable (see Table 1), which were significant in all cases (>0.5).


(1) $$\begin{array}{l} AVE=\frac{\sum_{i=1}^{k}\lambda_{i}^{2}}{\sum_{i=1}^{k}\lambda_{i}^{2}+\sum_{i=1}^{k}\mathrm{var}\left(\varepsilon i\right)} \end{array}$$

The calculation showed that all AVEs were significant (>0.5). In precise, AVEs values were between 0.61 (ETL) and 0.53 (SWB). These significant AVE values indicate high convergent validity for all variables.

**Table 1:** Convergent validity, composite reliability, and factor loadings.

	**λ**	**λ^2^**	**E-Variance**
**ETL**			
	0.84	0.71	0.29
AVE = 0.61	0.69	0.48	0.52
CR = 0.94	0.70	0.49	0.51
	0.73	0.53	0.47
	0.81	0.66	0.34
	0.84	0.71	0.29
	0.77	0.59	0.41
	0.86	0.74	0.26
	0.73	0.53	0.47
	0.79	0.62	0.38
**EBO**			
AVE = 0.60	0.80	0.64	0.36
CR = 0.91	0.83	0.69	0.31
	0.72	0.52	0.48
	0.70	0.49	0.51
	0.70	0.49	0.51
	0.76	0.58	0.42
	0.89	0.79	0.21
**SWB**			
	0.72	0.52	0.48
AVE = 0.53	0.77	0.59	0.41
CR = 0.81	0.71	0.50	0.50
	0.71	0.50	0.50
**ERS**			
	0.73	0.53	0.47
AVE = 0.56	0.76	0.58	0.42
CR = 0.88	0.71	0.50	0.50
	0.75	0.56	0.44
	0.84	0.71	0.29
	0.70	0.49	0.51

λ, factor loadings; CR, composite reliability; ∑λ^2^, sum of square of factor loadings; E-Variance, error variance.

Similarly, to calculate CR values for ETL, EBO, SWB, and ERS, we employed the formula in Equation (2). The standardized factor loadings were again the starting point to calculate CR values. We observed that the CR values were also significant in all cases (>0.7). Thereby, it was statistically established that CR was established in all cases in this survey (ETL = 0.94, EBO = 0.91, SWB = 0.81, and ERS = 0.88).


(2) $$\begin{array}{l} Compositereliability=\left(\left(\sum \lambda i\right)2/\left(\sum \lambda i\right)2+\sum \mathrm{var}\left(\varepsilon i\right)\right) \end{array}$$

### Model fit comparison

To see whether the original hypothesized four-factor model of this study fits well with the dataset of this survey, we made a model fit indices based comparison (for example, NFI, CFI), chi-square values/degree of freedom (χ^2^*/df)* and RMSEA values. To do this, we developed four measurement models (model 1 = one factor, model 2 = two-factor, model 3 = 3 factor, and model 4 = four-factor). This comparison was done to see which model produces superior model fit values. This is in line with the prior researchers. The outcomes of these four models have been summarized in Table 2. It can be seen that model 1 poorly fits the data showing that this should not be the appropriate model. Model 4 provides the evidence that it is the superior one because the model fit values were all superior for this model than all other models (NFI = 0.96, CFI = 0.96, χ^2^*/df* = 2.16, and RMSEA = 0.047). In precise, the change in χ^2^*/df (*Δχ^2^*/df*) values was between 0.84 (model 2 and 3) to 3.66 (model 3 and 4).

**Table 2:** Summary of model fit comparison.

**Model**	**Composition**	***χ^2^/df* (<3)**	**Δχ^2^*/df -***	**NFI (>0.9)**	**CFI (>0.9)**	**RMSEA (<0.08)**
4	(Original model) ETL, EBO, SWB, ERS	2.16	_	0.96	0.96	0.047
3	(Three-factor) ETL+ SWB, ERS, EBO	5.82	3.66	0.68	0.64	0.084
2	(Two-factor) ETL+EBO, SWB+ ERS	6.66	0.84	0.62	0.58	0.097
1	(One-factor) ETL+EBO+SWB+ ERS	8.13	1.47	0.49	0.48	0.157

### Correlations

We also assessed how the variables in this study covariate with each other. In this vein, the correlation (*r*) was observed between different pairs. The summary of the results has been given in Table 3. The analysis showed that variables co-vary with each other with different compositions (for example, positive, between ETL and ERS- and negative, between ETL and EBO-). Nevertheless, all values (positive or negative) were significant, which showed that correlations were significant. Further, as it can be seen from Table 3, no extreme case was observed (a case between 0.8 and 1), which rejects the possibility of multicollinearity. Last of all, we also verified the discriminant validity of variables (bold diagonal values in Table 3). It was observed that the discriminant value of a variable was higher than the *r*-values.

**Table 3:** Correlations and discriminant validity.

**Construct**	**ETL**	**EBO**	**SWB**	**ERS**	**Mean**	**SD**
ETL	**0.78**	−0.39	0.44	0.32	2.53	0.51
EBO		**0.77**	−0.26	−0.48	3.22	0.56
SWB			**0.73**	0.38	2.91	0.58
ERS				**0.75**	3.290	0.61

S.D., standard deviation; diagonal, discriminant validity values, p < 0.001. Bold values represent discriminant validity.

### Hypotheses evaluation

**Figure 1:**
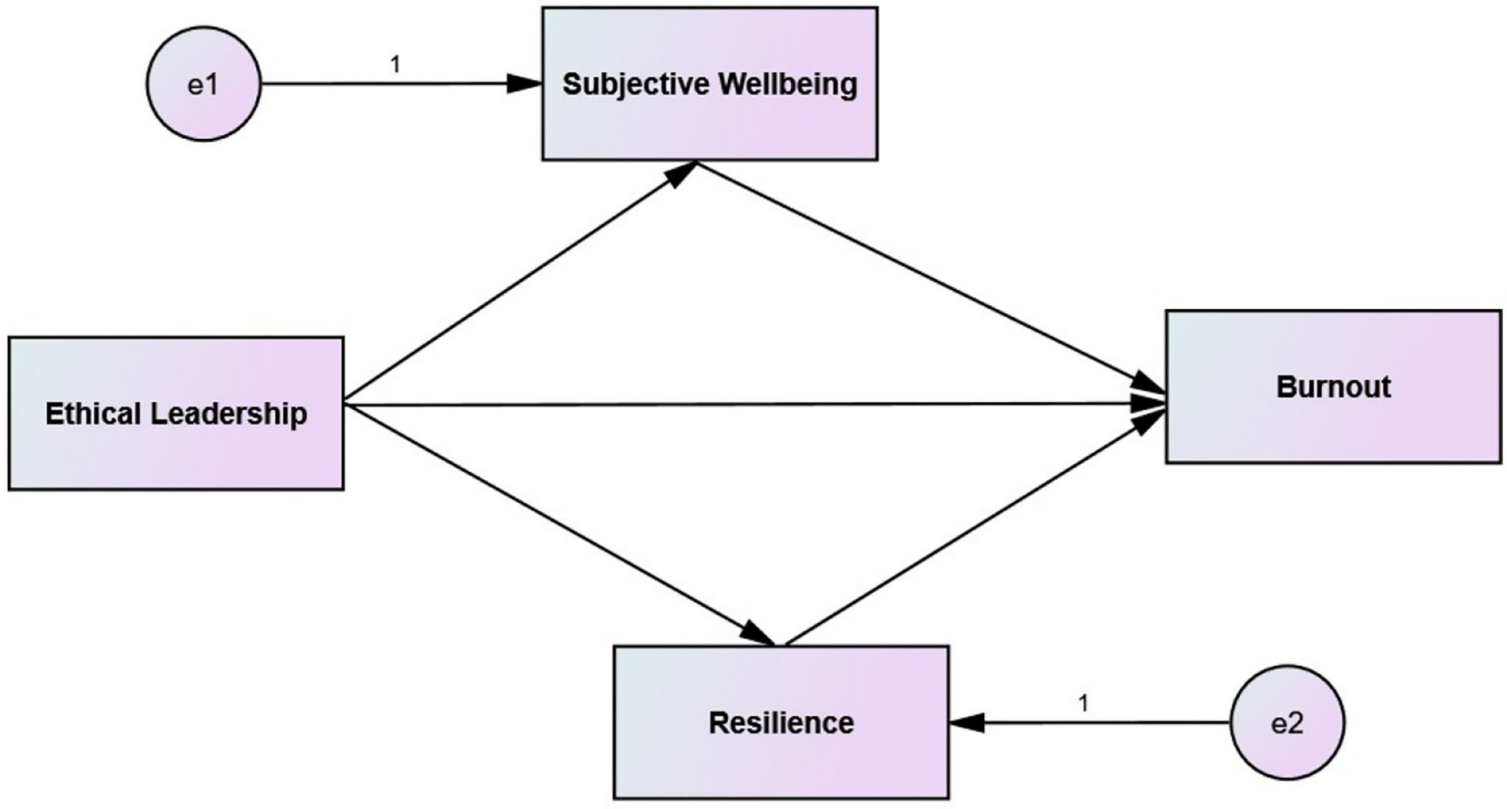
The hypothesized structural framework.

As a final point, we tested our hypothesized relationships with the help of structural equation modeling (SEM), a famous data analysis technique to analyze complex models (like in this study). We used the bootstrapping tool in AMOS to detect the significance of mediation effects by considering a larger bootstrapping sample (Ahmad et al., 2022b,c). First of all, we evaluated the direct effects between variables (for example, ETL → EBO, ETL → SWB, and ETL → ERS). These results have been presented in Table 4, which shows that three hypotheses were statistically significant (H1, H2, and H4). As an example, it was realized that ETL negatively predicted EBO (−0.41). This was significant (*p* < 0.05, and Confidence intervals were non-zero −0.589, −0.422). This finding was in line with the theoretical statement of H1, thereby confirming that the statistical evidence supported H1. The mediation effects were also significant, indicating that H3 and H4 should be accepted (ETL → SWB → EBO = −0.21; ETL → ERS → EBO = −0.17, *p* < 0.05 with non-zero confidence interval values). Figure 1 contains the hypothesized structural model of this study.

**Table 4:** Hypotheses results.

**Hypotheses**	**Estimates (SE)**	***t/z***	***p-*value**	**CI**
(ETL → EBO = H1)	−0.41 (0.05)	−8.20	****	−0.589, −0.422
(ETL → SWB = H2)	0.39 (0.06)	6.50	0.003	0.247, 0.482
(ETL → ERS = H4)	0.28 (0.03)	9.33	****	0.196, 0.310
Mediation effects (ETL → SWB → EBO)	−0.21 (0.02)	−10.50	****	−0.288, −0.159
(ETL → ERS → EBO)	−0.17 (0.01)	−8.50	0.005	−0.217, −0.112

CI, 95% confidence interval with lower and upper limits.**** means significant values.

## Discussion

The empirical findings support the theoretical statements of all hypotheses. Specifically, it was realized that an ethical leader negatively predicts employee burnout (β = −0.41). This is in line with the previous studies (Yang, 2014; Kaffashpoor and Sadeghian, 2020). A corporate leader with high ethical values is likely to enhance employees' wellbeing perceptions by solving their problems and by providing them with a flexible and positive work environment that provides employees with extra support and resources to improve their mental health, thereby reducing the likelihood of employee burnout (Franczukowska et al., 2021). Similarly, ethical leaders influence employees through their ethical conduct, which fosters their mental health and reduces burnout risk. Also, by referring conservation of resources theory, these results are justifiable because to recover from different negativities in a workplace, employees need contextual resources in the form of ethical leadership. Stressful working conditions are likely to build perceptions on the part of employees that their resources have been lost. This feeling of resource loss or insufficiency gives rise to the risk of burnout. From this perspective, corporate leaders could provide different resources to the employees, which limits the risk of burnout (Kanste et al., 2007; Arnold et al., 2015). Therefore, the role of an ethical leader is of pivotal importance in mitigating employee burnout in a certain hotel.

Another important takeaway of this research was to realize the important role of different psychological factors as mediators in explaining employee burnout as an outcome of ethical leadership. Especially, our research supports the theoretical assumption of H2 and H3 in which we expected that ethical leadership directly influences the subjective wellbeing of employees (H2), which then mediates between ethical leadership and employee burnout (β = −0.21). A positive link between ethical leadership and subjective wellbeing of employees has been discussed in prior literature repeatedly (Yang, 2014; Kaffashpoor and Sadeghian, 2020). Literature suggests that an ethical leader improves employees' mental wellbeing by focusing on their issues and facilitating them to accomplish different tasks (Li et al., 2014). Ethical leaders treat employees fairly, and when employees see fair treatment, it improves their mental health, which then fosters their subjective wellbeing, thereby supporting H2 (β = 0.39). An ethical leader is expected to provide his or her employees with optimal workplace environment by inspiring, building and maintaining an environment of trust and respect. Employees, as the receivers of such support and help from their ethical leadership, positively perceive such steps, which then thrive their subjective wellbeing. This is in line with past researchers (Sarwar et al., 2020). Employees with a higher level of subjective wellbeing have better mental health. Past researchers have also acknowledged the importance of employees' subjective wellbeing to reduce workplace negativities (Katana et al., 2019; Darvishmotevali and Ali, 2020). Even the mediating role of subjective wellbeing in leadership literature was also discussed (Hayat Bhatti et al., 2020). However, such studies did not explore the mediating role of subjective wellbeing in a hospitality context.

Lastly, our results also provided statistical grounds to accept the theoretical statements of H4 and H5. Indeed, ethical leaders improve employee resilience (H4) which mediates the negative relationship between ethical leadership and employee burnout (H5). Our study is in line with the findings of Brennan (2017), who mentioned that employee resilience is critical in predicting different employee outcomes in an organizational setting, including emotional wellbeing and professional success. Resilient employees are expected to deal with the risk of burnout by finding and arranging different personal and contextual resources. Moreover, employee resilience helps them to regain equilibrium after a stressful situation. To this end, the positive steps taken by an ethical leader in a hotel organization for the wellbeing of employees influence employees' cognition and satisfaction which then improve their resilience. Especially, the moral characteristics of an ethical leader improve the mental health of employees, thereby improving their resilience (β = 0.28). Our results are in line with previous researchers who indicated that ethical leaders could influence the resilience of their followers by building respect, consistency and trust which then influences their resilience (MacIntyre et al., 2013b). From a leadership standpoint, a leader helps employees to build a higher level of resilience by promoting employees' mindfulness and personal resilience plans (Sommer et al., 2016). Moreover, the ethical focus of an ethical leader enriches their level of trust and satisfaction, which leads employees to a greater resilience level. Hence, we provide statistical grounds to accept the mediating role of resilience between ethical leadership and employee burnout (β = −0.17).

### Implications

#### Theoretical implications

From a theoretical standpoint, this study advances the available literature on employee burnout from a leadership perspective in a hospitality context. Especially out research highlights the role of ethical leadership to mitigate the risk of employee burnout. Although the available literature acknowledges the role of ethical leadership from an employee burnout perspective, however, shockingly, previous literature neglected the hospitality sector as most of the studies were conducted in the healthcare sector (Okpozo et al., 2017; Franczukowska et al., 2021). To this end, our argument is that hospitality employees also face stressful situations and hence face a higher risk of burnout. Therefore, it was important to advance this debate from a hospitality perspective. Moreover, previously the phenomenon of burnout was explored in hospitality context but the role of leadership, especially ethical leadership, was not emphasized. For example, Harjanti (2019) indicated that burnout is escalating in hospitality, and he proposed that employee cooperation may reduce it. Similarly, Ayşegül and Erkan (2018) mentioned that quality of working life could significantly mitigate burnout risk in the hospitality sector. However, our research enriches the available literature by highlighting the role of ethical leadership in mitigating burnout risk among hospitality employees. Although current literature acknowledges the role of ethical leader from a burnout perspective, however, such literature is sparse in the hospitality context.

Further, we tend to enrich the available literature on enterprise management and employee burnout by proposing a robust model because the theoretical model of this study not only considers the direct relationship between ethical leadership and employee burnout but also considers the mediating role of different psychological and personal factors to explain employee burnout. Considering the complex nature of human behavior, we feel it was important to combine organizational, psychological and personal factors in a unified model, which, at least to our knowledge, was not investigated earlier. Specifically, our research extends the theoretical model by Schwepker and Dimitriou (2021), who did a wonderful study to explain the job performance quality of hospitality employees from an ethical leadership standpoint with the mediating role of job stress. However, these authors missed how ethical leaders could influence employee burnout, though these authors mentioned burnout in this study. Similarly, Dyrbye et al. (2020), in their recent survey, indicated how the manifestation of an ethical leader could predict burnout, however, they did not highlight the underlying mechanism of this phenomenon with the help of mediators (which we did in this study) to explain how and why ethical leaders improve burnout in an organization.

Furthermore, our study enriches the existing hospitality literature on employee burnout from a developing country standpoint (Pakistan), whereas most of the literature was carried out in developed countries. For example, Prentice and Thaichon (2019) investigated burnout in the hospitality sector of the USA. Similarly, Mansour and Tremblay (2018) surveyed employee burnout in the Canadian hospitality sector. Because, compared to developed countries, the enterprises in developing countries do not have adequate resources (infrastructure, facilities, and others), the studies conducted in developed countries may not reflect the case of hospitality employees' burnout in developing countries. Therefore, our research tends to fill this knowledge gap by investigating how ethical leaders could mitigate employee burnout in the hospitality sector of Pakistan, a developing South Asian country. Previously, such investigation was not carried-out in the hospitality context of a developing country.

#### Managerial implications

This study also provides some important practical implications for the hospitality sector of Pakistan. Considering the rising rate of employee burnout in this sector, our research attempts to mitigate this risk by promoting the ethical leadership style in hotel organizations. Specifically, our results will redound to the benefit to the management of a certain hotel to reduce the risk of employee burnout as an outcome of ethical leadership. The hotel administration needs to realize that the existence of an ethical leader improves the mental health and wellbeing of employees which in return reduce burnout risk among hospitality employees. By promoting this style of leadership, a certain hotel organization can effectively manage employee burnout. Therefore, the management of a certain hotel is required to arrange special training programs to promote ethical style of leadership among the managers.

On a further note, our research highlights the important role of different psychological and personality factors in reducing employee burnout. Given that employee resilience and subjective wellbeing could mediate between ethical leadership and employee burnout, this research suggests hospitality management foster these psychological and personality factors in an ethical leadership framework. The presence of an ethical leader creates a social bonding among employees with their leadership. Moreover, employees feel that an ethical leader focuses on improving their wellbeing and resilience. Employees with better wellbeing perceptions and a higher level of resilience are expected to face less risk of burnout. Hence ethical leader not only has a direct relation with employees' burnout, they also improve different psychological and personal factors of employees including wellbeing and resilience which explain burnout by creating mediating effects. Lastly, as was specified at the onset of this study that burnout is a challenge that relates to organizations, not the individuals, the leadership style of a hotel organization has a clear role in reducing employee burnout. Therefore, corporate leaders are responsible for protecting their employees against different workplace negativities, including the risk of burnout. Hence, we propose hospitality management to consider ethical leadership style for better and more effective organization management.

### Limitations and possible future directions

Firstly, this study collected data from two cities of Pakistan which limits the generalizability of this research. To deal with this limitation, we recommend including more cities from different provinces in future surveys. Secondly, we used non-probability sampling in this study. Though this sampling strategy has been extensively used previously, we believe a probability-sampling method is superior to predicting better causality. Therefore, we suggest using a probability sampling method for example simple random sampling in future studies to avoid the issues associated with non-probability sampling techniques. Lastly, this study considered only the hospitality sector from a burnout perspective. Recent evidence suggests that other service segments where employee-customer interaction is frequent also have a high employee burnout rate. For example banking employees also face stressful situations. Therefore, we suggest in the future, a comparative study may be conducted between other sectors to verify the role of ethical leadership from a burnout perspective.

## Conclusion

Admittedly hospitality is at a crossroads. While growth and development in this sector suggest a strong economic boom, the rising burnout due to stress, irregular working hours, and difficult working situation create a severe challenge for the management in this sector. Considering the negative consequences associated with employee burnout, it is pivotal to deal with this issue efficiently. Employees with burnout symptoms show the least level of energy to complete their job tasks at the one end, such employees also undermine customer loyalty because such employees are unable to provide quality services to the customers. All in all, burnout in any form has severed threats to a certain hotel organization. As a remedy to the level of management, we suggest promoting an ethical style of leadership to reduce employee burnout in the hospitality sector. An ethical leader not only provides employees with the necessary resources but he or she also keeps the morale of employees high by encouraging, supporting and facilitating them in difficult times. Therefore, we suggest hotel management promote this leadership style for effective organizational management. Similarly, the management of a certain hotel is also suggested to foster employee resilience and subjective wellbeing by arranging different personal resilience plans and workshops with a special focus on promoting these psychological and personality factors. To conclude, to prepare for the “black swan,” especially employee burnout, an ethical leadership style of management is a way forward for the hospitality sector.

## Data availability statement

The raw data supporting the conclusions of this article will be made available by the authors, without undue reservation.

## Author contributions

All authors listed have made a substantial, direct, and intellectual contribution to the work and approved it for publication.

## Conflict of interest

The authors declare that the research was conducted in the absence of any commercial or financial relationships that could be construed as a potential conflict of interest.

## Publisher's note

All claims expressed in this article are solely those of the authors and do not necessarily represent those of their affiliated organizations, or those of the publisher, the editors and the reviewers. Any product that may be evaluated in this article, or claim that may be made by its manufacturer, is not guaranteed or endorsed by the publisher.
